# Qualitative insights from a randomized clinical trial of a mother–child emotional preparation program for preschool-aged children

**DOI:** 10.1186/s40359-023-01288-y

**Published:** 2023-09-01

**Authors:** Elizabeth S. Markowitz, Malia C. Maier, Robert J. Ludwig, Judy Austin, Anna M. Maybach, Marc E. Jaffe, Martha G. Welch

**Affiliations:** 1 Department of Pediatrics, Columbia University Irving Medical Center, New York, NY USA; 2 Mailman School of Public Health, Columbia University Irving Medical Center, New York, NY USA; 3 Department of Psychiatry, Columbia University Irving Medical Center, New York, NY USA; 4 Children’s Learning Centers of Fairfield County, Stamford, CT USA; 5 Department of Pathology and Cell Biology, Columbia University Irving Medical Center, New York, NY USA

**Keywords:** Emotional connection, Autonomic co-regulation, Randomized controlled trial, Calming cycle theory

## Abstract

**Background:**

Early life stress and adversity conveys risk for emotional, behavioral, and developmental disorders. To address this risk in the preschool population, Mother–Child Emotional Preparation (MCEP) was tested as an in-school dyadic intervention for facilitating mother–child emotional connection through mother–child *calming cycles*. In a computer-generated block randomized controlled trial enrolling preschool-aged children and their mothers, in partnership with an early childhood learning center, we at Columbia University Irving Medical Center tested effects of MCEP across multiple domains. Within this RCT we designed a qualitative sub-study to understand how MCEP aligns with *calming cycle theory* and its impact on mothers and the mother–child relationship.

**Methods:**

A qualitative researcher observed 14 group MCEP sessions consisting of nurture specialists facilitating reciprocal calming interactions through shared emotional expression between mothers and their preschool-aged children. We conducted two waves of participant interviews in English or Spanish, per participant preference. Participants (*n* = 8) were majority Hispanic at or below the federal poverty level. Group session observations were coded and analyzed for frequency, co-occurrence, variance by session, and alignment with calming cycle theory, incorporating demographic variables and attendance. Interview transcripts were translated from Spanish to English if needed, then coded and analyzed using thematic analysis.

**Results:**

Qualitative analysis revealed mothers’ experiences of MCEP. Data demonstrated that calming position and emotional expression were mutually supportive, and that barriers to connection were calming cycle entry-points, not barriers. At the group level, supported by nurture specialists, fellow participants helped each other progress through calming cycles. Moreover, MCEP adapted to meet individual dyad needs, and mothers described its far-reaching impact.

**Conclusions:**

Qualitative methods show that MCEP helps mother–child dyads emotionally connect through the calming cycle and fills a gap in early childhood education services. This study generated insights for quantitative studies and suggested implications for MCEP dissemination.

**Trial registration:**

ClinicalTrials.gov, NCT03908268, Registered April 9, 2019—Retrospectively registered.

**Supplementary Information:**

The online version contains supplementary material available at 10.1186/s40359-023-01288-y.

## Background

Early life stress and adversity conveys risk for emotional, behavioral, and developmental disorders. To address this risk in the preschool population, Mother–Child Emotional Preparation (MCEP) was tested as an in-school dyadic intervention for facilitating mother–child emotional connection through mother–child *calming cycles*. Based on decades of implementation by Martha G. Welch MD, and then through randomized control trials with collaborators, our group in Pediatrics at Columbia University Irving Medical Center (CUIMC) tested the therapeutic potential of facilitating parent–child emotional connection, first in a preterm population and here in a preschool population. Now there remains a need for qualitative research describing this intervention in participants’ own words. Here, in a sub-study of the preschool Randomized Controlled Trial (RCT), we enable participating mothers to introduce parent voices to early childhood professionals treating struggling parents. In doing so, we elucidate the impact of group sessions on participants, analyze factors possibly moderating impact, and conclude with key takeaways for designing and implementing MCEP interventions for dissemination.

MCEP was developed at Children’s Learning Centers of Fairfield County (CLC) in collaboration with our group at CUIMC [1]. The intervention is based on the hypothesis that a daily cycle of mother–child *autonomic co-regulation* can facilitate emotional connection at the autonomic nervous system level. This connection can lower stress levels of both at home and improve child behavior and learning in the classroom. Repeated mother–child *calming cycles* during MCEP aim to establish emotional connection and co-regulation to improve outcomes across multiple domains. *Calming cycle theory* is the theoretical basis of this intervention [2–6].

The current qualitative literature examining similar interventions is lacking the depth and breadth of insight afforded by a dual interview and participant-observation lens. Schuster et al. (2018) [7] and Brandão et al. (2019) [8] used interviews to explore participants’ experiences but did not consider efficacy. More recently, Chaudhry et al. (2023) [9] used quantitative measures combined with qualitative interviews with seven participants in a maternal-child play intervention to explore methodology and its “practical barriers,” in addition to its effect on child development, parenting practices and mother–child relationships. While the study did incorporate interviews, like ours, it did not involve direct observation.

Combining multiple qualitative data sources is scarce in the group therapy literature. So’s (2019) [10] triangulation of interview transcripts, participant journals, and audio-recorded sessions to examine student experiences with group music therapy shows the analytical benefit of capturing different dimensions of the same phenomenon qualitatively. Synthesizing MCEP observations with participant interviews affords a richer, more nuanced understanding of MCEP.

MCEP’s emphasis on relational health also differentiates it from existing group intervention studies. Scope et al.’s (2012) [11] synthesis of postnatal depression group interventions found that treatment enabled women to develop better relationships with their infants—but the intervention solely targeted the mother. Mother-infant psychoanalysis does involve both mother and infant, but this method addresses emotional distress through infant-therapist and mother-therapist interactions rather than direct mother-infant interactions [12]. Dyads are engaged individually, leaving participants to figure out when and how to apply their newfound skills to their relationship. MCEP is therefore the first intervention to date impacting the mother–child relationship at the autonomic nervous system level through direct emotional engagement in a group school-based setting. Moreover, it is experiential, not didactic, with direct mother–child engagement, instead of cognitive, with therapist-patient instruction.

This observation-interview lens also elucidates how MCEP for preschool-aged children aligns with calming cycle theory. Understanding this alignment is crucial because calming cycle theory underpins the design and implementation of MCEP. Based on quantitative results [13], we know that MCEP is effective in changing child behavior at home and at school, and at facilitating mother–child emotional connection. Nevertheless, a deeper understanding of the mothers’ point of view and feelings is important for refining MCEP training and implementation.

### Intervention methodology: the calming cycle

**Fig. 1:**
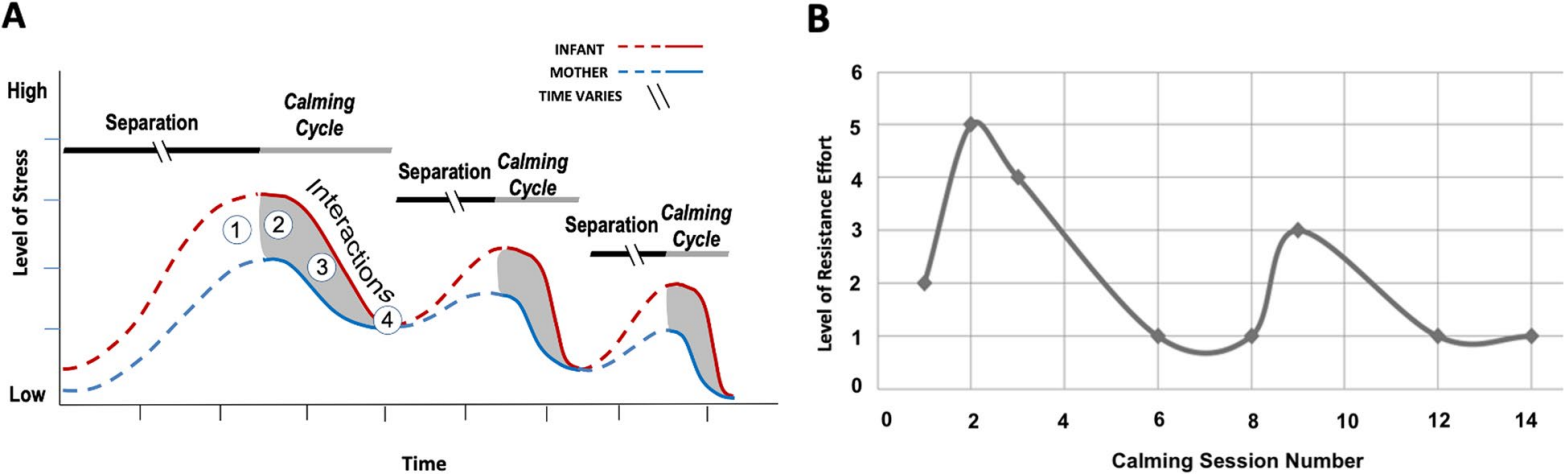
The Calming Cycle theoretical construct. Panel **A** illustrates the four phases of calming cycle: 1) separate mother and child distress, 2) mutually shared distress, 3) mutual resolution of distress, and 4) mutual calm that include observable periods of eye contact and/or physiological calming. Note that over time repeated calming interactions between mother and child lower levels of stress in both the mother and the infant and with progressively less duration of interaction. Panel **B** plots the observed level of resistance effort over repeated calming sessions. Note the similarity in the shape of the two graphs, which supports the idea that the intervention lowers levels of stress and resistance to closeness

The Welch “calming cycle” method grew out of four decades of clinical observations. It views the mother–child relationship as interconnected and fluid, cycling through autonomic states of dysregulation and co-regulation [4, 5]. The learning mechanism is theorized to be “functional” Pavlovian autonomic conditioning [3]. In contrast with attachment therapies, where the mother–child relationship is viewed as a product of psychological “trait-based” attachment and “enduring” bonds [14], emotional connection is state-based, the product of child and mother’s autonomic nervous systems, and can be changed at any moment by authentic shared emotional expression. In a calming cycle, when a child demonstrates a dysregulated state—crying and resisting eye contact—the mother expresses her own upset until they reach calm together. After this cycle, the mother–child pair spontaneously maintains eye contact, speaks softly, demonstrates physical affection, and feels reciprocal joy. To reconnect emotionally after separation or conflict, a mother–child pair learns to progress through the calming cycle’s four stages (Fig. 1a): 1) separate mother or child distress, 2) mutually shared distress, 3) mutual resolution of distress, and 4) mutual calm.

MCEP sessions began with greetings between participants, introducing existing participants to any new pairs joining the group that week. Then mothers situated their children on their laps facing them in the *calming position* and were encouraged by nurture specialists to *express* directly to their child *what they are each feeling emotionally*. If struggling to speak emotionally, nurture specialists offered prompts such as “tell your child what you love about him/her” or “talk about something that hurt your feelings.” The child may *resist* this closeness and become dysregulated. This distress often prompts an emotion in the mother that she is encouraged to express to the child. The processing of that exchange is the mutual distress phase. To initiate the third phase, the mother expresses how her child’s behavior makes her feel, sometimes through tears. This exchange orients the child to the mother as the child feels her distress. The mother may engage in behaviors like touch, emotional expression, soothing, and when possible, *eye contact,* to begin calming the child. Positive responses from the child, such as tender attention to the mother, initiate the final phase of mutual calm, characterized by sustained mutual eye contact, warm, open communication, and relaxation and reciprocal pleasure in each other’s presence. If the child continues rejecting the mother, another calming cycle begins. It may take multiple calming cycles or many sessions to (re)establish emotional connection. When dyads’ emotional expression abilities were blocked, or their emotions were very intense, other participants sometimes paused their own sessions to encourage a specific pair.

## Methods

To enrich the quantitative assessments already employed in the parent RCT, we used an inductive grounded theory qualitative design with hypothesis coding to answer impact questions: 1) the impact of MCEP on the mother–child pair in the mother’s own words and 2) MCEP alignment with calming cycle theory, as per observation of eight study sessions and interviews with participants immediately following completion of the study and at two-month follow-up.

### Population and participants

Our CUIMC group partnered with CLC to embed MCEP into CLC’s program; this study was conducted as a part of NCT03908268 (ClinicalTrials.gov; registered April, 9, 2019; retrospectively registered; https://clinicaltrials.gov/ct2/show/NCT03908268). CLC is a community-based multisite nonprofit preschool in Stamford, Connecticut. The program is one of only 13% of such programs in the country accredited by the National Association for the Education of Young Children. CLC serves ~ 1,000 children ages six weeks to five years and operates 10 h per day, five days per week and 51 weeks per year. A large percentage of families are immigrant, with English as their second language. Eighty-seven percent of families are considered low-income and eligible for free or reduced school lunch [15]. CLC focuses on child social and emotional development as well as parent engagement. CLC’s curriculum incorporates a range of subjects including pre-literacy, pre-numeracy, creative play, health, wellness, and nutrition.

Mothers and their children aged two to four-and-a-half were eligible for this study. Participants were randomized (1:1) to either the MCEP plus Standard Care (SC) or the SC group from December 2018 through December 2019. MCEP mothers were given 16 weeks to complete eight two-hour sessions of MCEP at three CLC sites. MCEP sessions consisted of nurture specialists facilitating reciprocal calming interactions between mothers and their children.

### Sampling for the qualitative component

Qualitative data were collected from one intervention group in the study, at one of the three CLC sites. The study site was chosen for logistical convenience. Of the nine mother–child pairs who consented to participate, one withdrew due to relocation. Participants at the selected CLC site were consented in-person to additional participation in the qualitative study at the time of consent to the RCT. They gave an additional signature on an RCT consent form with a qualitative section. To prioritize ethical considerations, participants’ ability to withdraw from the qualitative or RCT study was emphasized. No additional compensation was given for this sub-study.

### Data collection

Following an inductive grounded theory approach, ESM unobtrusively observed all MCEP sessions to capture the in-vivo experience of the intervention. Participants were interviewed immediately after their final session to capture their initial responses to the intervention in their own words, and then again two months later to capture the effect of the intervention at the time of their final follow-up with the RCT. Study sessions took place in a classroom cleared of furniture and set up with an area rug, large pillows, and hallway window shades pulled down for privacy.

Two nurture specialists, a study coordinator, and ESM were present during all MCEP sessions. ESM audio-recorded, observed, and took notes during the group’s 14 sessions, of which each dyad attended eight (or in two cases, seven). AMM transcribed the recordings and added verbatim dialogue to observation notes if missing. ESM conducted semi-structured in-person interviews (Supplement 1) in English or Spanish (per participant preference) with all eight participants immediately after the intervention (28–68 min duration) and with five participants via phone at the final assessment time, on average two months after the first interview (~ 30 min duration). Initial interviews took place in person in the study session room or a neighboring private classroom, with shades drawn for privacy. Interview transcripts were translated from Spanish to English by MCM, who is fluent in Spanish. ESM verified understanding of participants’ reflections at multiple points during both interviews, rather than return transcripts to participants, some of whom were not literate.

**Fig. 2:**
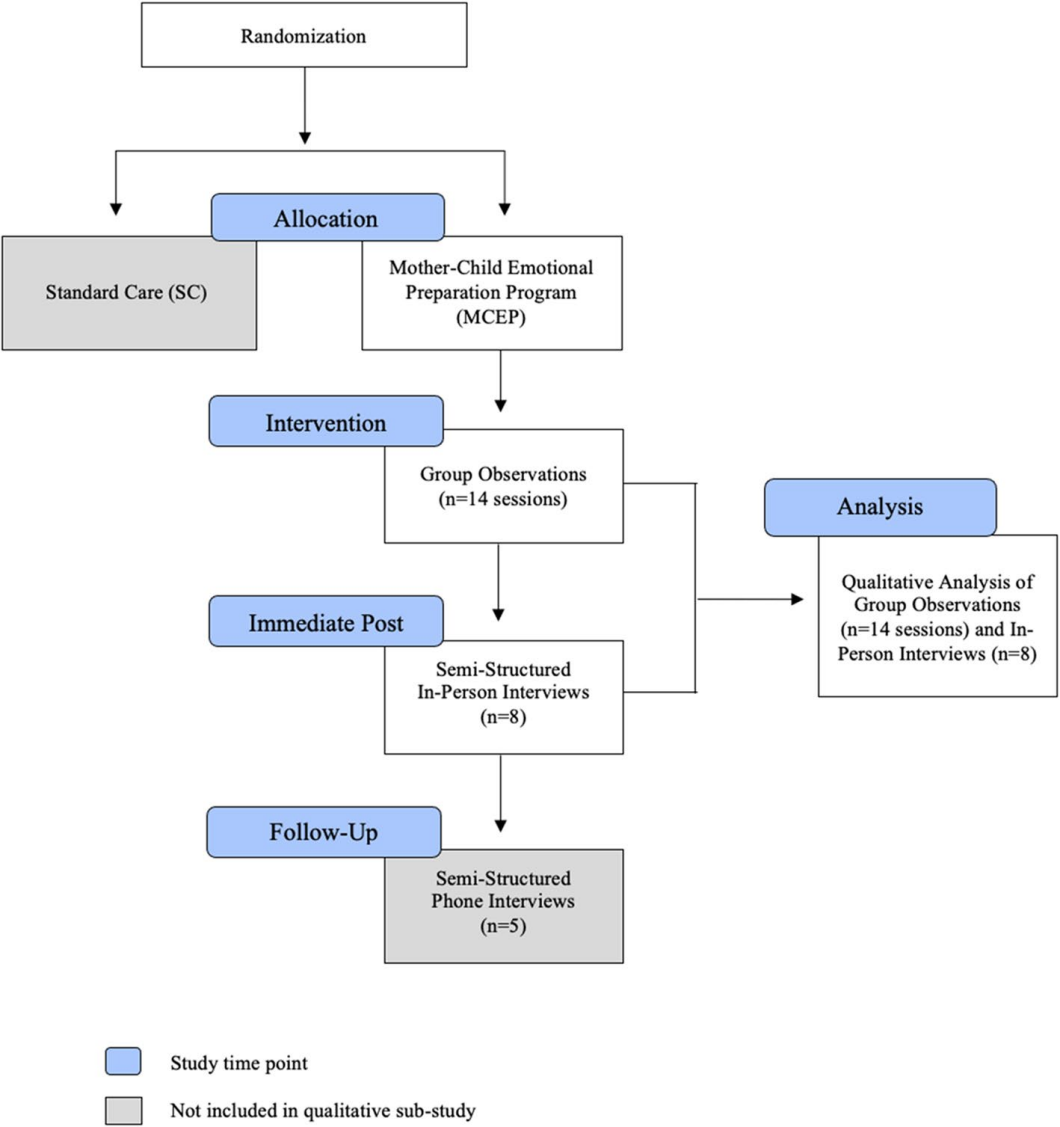
Qualitative sub-study workflow

Files were password-protected and de-identified. File access was granted only to those involved in data analysis (ESM, MCM, and AMM). The study design did not include pilot-testing because it was part of an ongoing RCT. Figure 2 summarizes data collection and analysis workflow.

### Data analysis

Group session and interview transcripts were coded by ESM and MCM, respectively. Initial codes were guided by the research questions [16, 17].

Group observation central concepts were shaped into a codebook by ESM and AMM through two initial codings and follow-up discussion. ESM and AMM each devised codes, discussed points of difference, agreed on categorization, and then coded again using the mutually-constructed codebook. Group observations and final codes were then imported into *Dedoose* (version 8.2.14) and analyzed for frequency, co-occurrence, variance by session, and alignment with calming cycle theory, incorporating demographic variables and attendance.

MCM devised the codebook used for interview analysis. Key themes were identified during translation, and initial codes were refined after discussions with an independent colleague. After two rounds of coding, thematic analysis was performed.

### Validity

Several measures were taken to strengthen “credibility, authenticity, criticality, and integrity,” identified by Whittemore et al. as primary validity criteria in qualitative research [18]. Credibility was increased by outsourcing the bulk of transcription to an independent company (Datalyst), and the remainder to a team member not involved in analysis. Further, the two authors involved in data analysis performed inter-coder agreement exercises with both codebooks. Authenticity was enhanced by including interview excerpts from all eight participants, as well as consulting the recordings whenever precise meaning was in question. Criticality was enhanced by discussing discrepancies in passage length coded and including only surrounding context essential for meaning in second-round coding. Integrity was ensured by supporting conclusions with verbatim quotes and code frequency statistics.

ESM and MCM further improved the validity of findings by accounting for their “positions” as researchers [19]. ESM’s observations may have influenced group interactions. An expectancy effect [20] may have resulted during interviews, whereby interviewees perceived and subconsciously confirmed the researcher’s hypotheses, especially as the participants grew familiar with ESM over the course of up to 8 sessions and two interviews. Prior to the study, no participant-researcher relationships existed. To minimize expectancy effect, the study goal of understanding participant perspectives was restated prior to interviews.

ESM identified and deconstructed subjectivity elements. Collecting data gave her an intuitive sense of code definitions, which needed to be more explicitly stated for the second coder. She was also the sole observer and interviewer. Her prior fieldwork for her MSc in medical anthropology may have stimulated a search for cultural or socioeconomic rationales for emotional connection differences amongst dyads. Her experience in anthropology may have narrowed the lens of study design. The research team discussed these potential biases and sought to account for them in the analytical process, with AMM and MCM adding contrasting perspectives for codebook generation and analysis of observations and interviews respectively.

MCM identified the following subjectivity issues: her personal experiences as a childcare provider for families with varying levels of emotional connection; professional experiences with parent–child dyads in the US and Latin America; and public health coursework for her MPH that favors responsive care to enhance child development. This may have led to postulations about the intervention’s positive impact, as well as assumptions about parenting approaches based on mothers’ cultural backgrounds.

To minimize the intrusion of these subjectivity factors during analysis, both researchers acknowledged initial hypotheses and prior knowledge while generating codes [21]. Interview analysis was blind, and neutral codes were applied to equally account for positive, negative, and neutral participant perceptions of MCEP. Frequent discussions with study staff and Columbia University faculty broadened perspectives and audited biases.

## Results: MCEP through a qualitative lens

Study participants were 8 mothers, ranging in age from 30–42, and their children aged 2–4.5. Participants were majority Hispanic and at or below the federal poverty level (Table 1) and enrolled in CLC of Stamford, CT.

**Table 1:** Demographic characteristics of eight MCEP mothers

Characteristic	Mothers *n* = 8 No (%)
Age median (range)	33 (30–42)
Ethnicity and Race	
Hispanic, White	1 (12.5%)
Hispanic, Race not specified	4 (50%)
Hispanic, Prefer not to answer	1 (25%)
Non-Hispanic, Asian	1 (12.5%)
Non-Hispanic, White	1 (12.5%)
Education	
College (Bachelors)	1 (12.5%)
College (Associates)	1 (12.5%)
Some College	3 (37.5%)
Primary School	3 (37.5%)
Household income, median (range)	$30,000 ($18,000-$100,000)
Receives state or federal assistance	
Yes	4 (50%)
No	4 (50%)

### Calming position & emotional expression function in tandem, tailored to each dyad

**Fig. 3:**
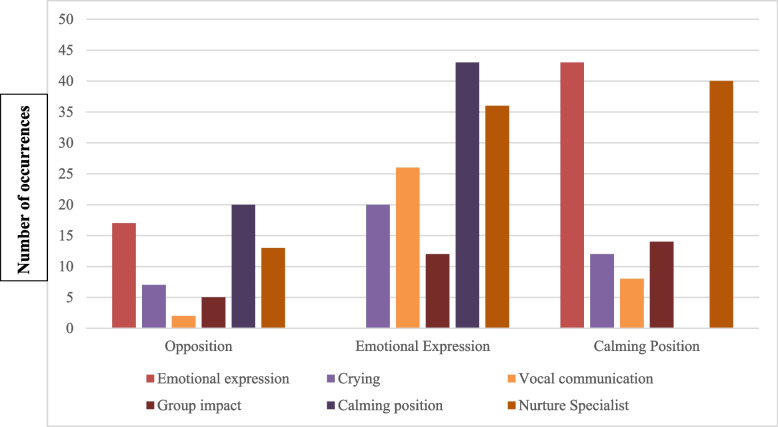
Observation code co-occurrence

Analysis revealed the importance of mother–child calming position and emotional expression. These themes most frequently co-occurred (Fig. 3), supporting the notion that positioning the child on the mother’s lap face-to-face facilitates emotional expression (and vice versa), and emotional expression facilitates emotional connection (and vice versa).

**Fig. 4:**
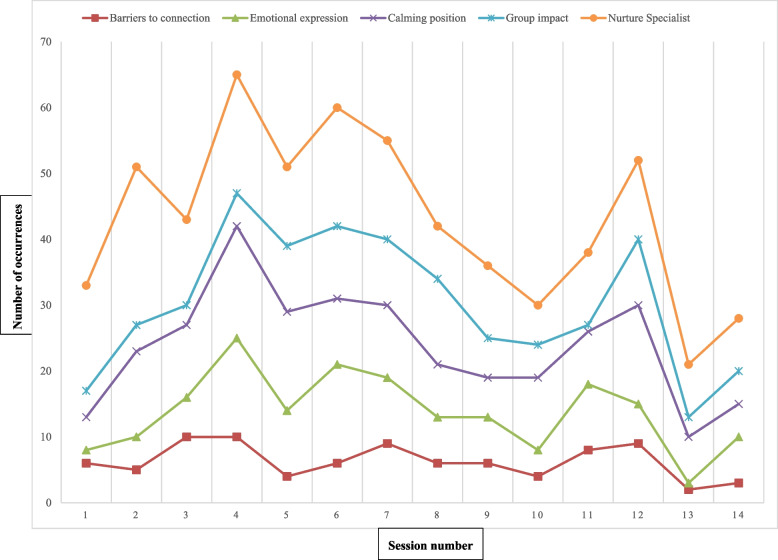
Selected observation code frequency by session

Analysis showed that emotional expression, featuring vocal communication and sometimes crying, peaked when participants were in the calming position (Fig. 4). The more the pair attended sessions, the more they vocally expressed their feelings to each other.

These findings support calming cycle theory’s view of crying as a spontaneous release of the full range of feelings prompted by dyadic disconnection. It appears to be useful but not essential for establishing emotional connection.

Crying was most commonly an expression of child dysregulation during the separate distress phase of the calming cycle. Of the 27 crying periods *during* calming cycles, 25 featured children, and two featured mothers. One example:

L(mother)*^1^ starts crying. Nurture specialists 2 and 3 urge her to show her child her tears.

NS 2: “She needs to see your tears. Don’t cover your face.”

NS 3: “K*, look at mommy! Mommy’s sad.”

…

L: “I don’t know what to do, when she wants something she goes to [her] dad, because he doesn’t like to see her cry. I can’t afford any more energy.” [Observation 12]

The mother’s (L’s) tears expressed her hurt, frustration, and exhaustion. Although K did not respond immediately, 30 min later, L leaned in to kiss her child, who kissed back. The next session, this dyad reached connection sooner, joyfully emotionally expressing together for the entirety.

L’s tears helped K recognize her behavior’s effect on her mother. Tears may be effective due to their inherent communication of deep feeling. For mother–child dyads stuck in the “individual distress” phase, asking the mother to express her feelings about, for example, dysregulated behavior may help the pair progress to resolution. Additionally, mothers crying during the concluding group discussion when reflecting on particularly difficult moments, elicited support from other mothers.

Another important element of emotional expression is the tenor, volume, and prosody of speaking—often coupled with intimate physicality. Speech is “soft, soothing” [Observations 3, 4 5, 7, 14] and often “whispering” [1, 3, 4]. Dyads sing together [Observations 1, 2, 3, 4, 5, 6, 13, 14], rock back and forth [3, 7, 13], and talk closely and cozily [1, 9]. For example, the researcher noted F* and R*, after emotionally connecting, “slipping easily into soft chatter” [Observation 7] and A* and J*(child) “pressed together, talking animatedly” with A “stroking J’s face” [Observation 1].

These interactions describe the elements of connection codified in the RCT’s primary outcome measure, the Welch Emotional Connection Screen (WECS). This brief screen assesses emotional connection in terms of parent–child facial communication, mutual attraction, sensitivity/reciprocity, and mutual vocal communication [22]. Research on the universality of facial expressions, the link between facial action and the autonomic nervous system, and the processing of dissonant facial expressions and tones suggest that matching facial expression and tone to sentiment optimizes neurological and autonomic processing time of another’s emotions [23–26].

For D*, the emotional expression component of MCEP helped her relinquish long-standing maternal guilt through apologizing. At the start of group sessions, D was ashamed over her harsh disciplining of her child B* years prior (“I hurt him, I hit him”) and lack of quality time (“I didn’t spend time with him”) during a period of postpartum depression from B’s birth through his toddler years. Apologizing to her child catalyzed their emotional connection. D later reflected on this moment in her interview:

“...when B was looking at me in the eyes like this…it was as if I started to understand him a little better…why he was a little distant from me…it was that I didn’t dedicate time to him, because I had never asked him for forgiveness.”

In subsequent sessions, D and B were physically affectionate and emotionally expressive. B was observed “kissing his mom…” and seeking skin-to-skin contact [Observation 10]. D’s apology alleviated her feelings of guilt and shame, giving way to emotional expression.

In session, children repeatedly sought contact with their mothers’ skin. The maternal-child benefits of skin-to-skin contact are documented mainly for preterm infants and include “cardiorespiratory stability” [27] and decreased risk of postpartum depression [28]. Interestingly, these interactions featured male children and their mothers: S* hiding his face under mom’s shirt [Observation 1], and B resting his head on his mother’s chest and melting into her shoulder [Observations 1 & 2].

This contact-seeking contrasts to the boys’ initial resistance to their mothers’ attempts to engage. In the first few sessions, it took mothers of the two boys relative to those of the six girls more effort to position their child to sit facing them. Boys were more physically aggressive towards their mothers. Large cross-cultural studies show higher levels of physical aggression but not “relational aggression” (e.g., between a mother and child) as “normative” among boys [29, 30]. In the case of these two boys—but not the six girls—there was relational aggression prior to treatment, which resolved according to post-treatment interviews. This observation (Fig. 1b) supports calming cycle theory (Fig. 1a), which predicts that the amount of relational aggression (i.e., discomfort and distress) will decrease with repeated calming cycles.

### Barriers to connection: obstacles as springboards

Multiple barriers to connection emerged initially as obstacles to reaching emotional connection. From low to high frequency, these included: social barriers (3 instances), lack of time at home (4), shyness (5), understanding the intervention (10), playing/silliness (17), and child opposition to maternal closeness (40). Though labeled “barriers,” we used them as tools for initiating calming sessions.

An example of a social barrier is a social network ignoring a health struggle. D explained: “In our churches, we don’t talk about depression because we don’t have a psychologist” [Observation 10]. This “cultural” barrier is more psychobiological (depression acting on the mother) and socioeconomic (inability to pay for treatment). D’s depression had resolved prior to enrollment in MCEP.

D’s socioeconomic risk factors remained. A Central American immigrant working as a housekeeper, she reported a total household income slightly above the federal poverty level. Limited income and variable work schedules, especially combined with immigration-related stressors, made it difficult for such mothers to set aside consistent time to connect with their child. These factors are associated with maternal depression and anxiety [31]. In turn, the depressed mother has a “compromised” ability to respond to her child, and the child is at higher risk for “disturbances in behavior and psychological functioning” [31]. Calming sessions helped D identify her lingering shame as a barrier to connection and supported her in establishing connection with B through emotion-infused apologizing.

Barriers can be used as entry points into the calming cycle. For example, E* shared that her upbringing discouraged the expression of strong feelings of anger or upset. Through MCEP, she saw the value of expression and began expressing herself to her child. When, for example, E cited lack of time at home as a barrier, it was part of a statement about the benefit of group time: “I’m really enjoying the time here…I’m busy, I have a lot to do. I have two more children at home…” [Observation 3].

Nurture specialists use calming session obstacles as avenues for engagement. When a mother reported shyness as a barrier in her relationship with her child, a nurture specialist encouraged this mother to “talk about how hard it is for her to be with other people, and how shy she [child] gets” [Observation 1]. In later sessions, this shyness became the focus of emotional expression. At the end of the intervention, the mother reflected primarily on the child’s increased sociability. When addressed directly by the mother–child pair, barriers serve as launchpads into a calming cycle.

The idea of a “barrier” as a path to emotional connection is clear in moments of “opposition.” Since opposition to the calming position or to emotional expression often characterizes the first phase of the calming cycle, it is not surprising that opposition is the most observed barrier to connection. A child may whine “let’s go, let’s go” [S Observation 9]; cry [K Observation 6]; or lean away [N* Observation 3]. The synergy of the calming position and emotional expression converts opposition from a connection barrier into the first phase of the calming cycle. It makes sense, then, that “opposition” was most visible in dyads’ first sessions, when they were learning how to enter calming cycles (Fig. 4). The low frequency of insurmountable blocks to emotional connection supports the idea of MCEP as effective independent of social and cultural forces or individual “temperament.”

### Group effect: participant and nurture specialist support

Nurture specialists facilitate the intervention. They help dyads process unresolved emotions in parent–child relationships and then embrace the calming cycle that ensues from this genuine sharing of emotion, evidenced by the co-occurring peaks of nurture specialist engagement and calming cycles in initial sessions (Fig. 4). Figure 4 shows that five selected themes have matching peaks and valleys by session, termed “co-occurrence”; this indicates that these elements of the calming cycle act in parallel, and possibly in concert.

Nurture specialists also highlight challenges in either the child or mother’s behavior that affects connecting. In one instance, a nurture specialist encouraged E to address her child’s standoffishness [Observation 5]. E later identified this interaction as a turning point:

“I said, well, I think that’s why I came, to be able to liberate myself of this a little…that day we talked about it. From that day, [H*] really changed her way of being, a lot.” [Interview 1]

This nurture specialist similarly encouraged C* to express her frustration at her child [N]’s clinging and shyness. C explained to N that she wants her to say ‘hello’ to people:

“Nothing happens when you say it! It’s not that hard. Look at me! Look at me!” [Observation 6]

The mothers were emotional and perhaps embarrassed when their child’s behavior was discussed, which further encouraged them to tell their child how the behavior was negatively impacting them. In both situations, the mother’s desired outcome of increased child sociability was achieved. C reflected on the importance of this encouragement during her post-group interview:

“NS 2, she was the one who made me realize more or less N’s problems, and she made me realize that there was a way I could make N change her thinking, talking to her about my feelings…it worked really well.”

A few participants were defensive in response to nurture specialist feedback. For example:

NS 1: “She looks sad. Look at her face. She looks sad.”

A: “I don’t think she’s sad...” [Observation 3]

The following week, the mother reported that she had spent days “practicing, talking, and sharing more” with her child—demonstrating the ultimate efficacy of the session [Observation 4].

Participating mothers are an organic source of mutual support given their shared experience. MCEP views fellow group members as a source of empathy, positive reinforcement, and modeling of successful calming cycles. All of these roles were supported by the data. L noted that the group validated her parenting experiences: “…it’s nice to know I’m not the only one having experienced one of those things” [Interview 1]. E similarly reported feeling less worried about problems she was facing at home:

“I have become a better person. Some things that I thought were bad, in reality they weren’t…listening to the experiences of the other moms…it’s like I released a little of the burden.” [Interview 1]

D and L sat next to or across from each other during three consecutive sessions. In their second neighboring session, after L’s particularly difficult exchange with her child D offered L empathy—a memory of how difficult it was for her when her child B would act up in public [Observation 5]*.* In her post-group interview, L highlighted this exchange as comforting and encouraging. Proximity may increase the potency of dyads’ emotional support of one another due to ability to read body language. Such participant-participant support underscores the value of the group model, consistent with prior research on group therapy [32, 33].

Analysis of the “group effect” demonstrated that MCEP adapts to the various needs of each dyad. D’s primary struggle was guilt from prior experiences of mothering during postpartum depression. The intervention helped her identify this as a source of disconnection and work through her feelings. A was alone in the country because no immediate family came with her when she immigrated five years ago [Observation 8]. Through MCEP, A connected with fellow mothers and nurture specialists and gained the sense of community she craved. According to C and N, with nurture specialist and group member support, they achieved the helpful interdependence that had been escaping them. Furthermore, L was able to finally express her pain to her child. Both dyads achieved mother–child emotional connection [Observation 12]. MCEP participant and nurture specialist support is naturally tailored to dyadic needs, since the entry point of a calming session is a dyad’s interactional challenge.

### The nuanced impact of MCEP: from good enough to better

F, the mother with the highest household income and education level, felt she had a “good enough” relationship with her child. One nurture specialist repeatedly emphasized the importance of discussing a sensitive topic, even if not pressing, to practice emotionally connecting in response to upsets that might arise. F maintained her skepticism that the intervention might be “provoking upset” in the group sessions. “I’m not a crier” she added at one point, offering her temperament as a reason for not participating in deep emotional expression [Observation 9]. Even when observing the marked transition in emotional connection for another dyad in one of the last sessions, F did not change her opinion on the utility of the intervention for her relationship with this one child of her four:

“I’m not saying I’m the wrong candidate, I’m just not sure I need the intervention, you know?” [Interview 1]

F’s opinion that she was not the right ‘candidate’ again raises the question of whether the group MCEP model is universally applicable. Interestingly, in her second interview, F admitted another child of hers might stand to benefit from the intervention. In any case, F continued to participate in the study (despite no consequences for dropping out) and returned for the follow-up interview. During this final interview, F reported having used MCEP principles to discuss emotions with her four children when a close relative died. Another participant, G*, noted that while she and her child had always shared moments of affection, these interactions became “something sweeter” after the sessions because they had a stronger connection.

These more subtle examples of MCEP’s impact show that the intervention is effective depending on each dyad’s willingness to engage, progressing relationships along a spectrum from instrumental care to the daily practice of emotional connection.

### Participant perspectives: MCEP’s effect beyond the group

Post-group interviews elucidated MCEP’s impact beyond the group. Three themes surrounding developments in the mother–child relationship emerged: changes in parent behavior, changes in child behavior, and improvements in the dyad’s emotional connection. These themes highlight the reciprocity of the mother–child relationship.

Through MCEP, many mothers developed a new form of communication with their child. For mothers who had been unable to assert themselves, this meant learning to speak to their child with more emotional expression and the authority that comes with it. In G’s case, this meant adopting an expression more appropriate to the moment:

“...now I don’t laugh, I stay serious looking at her… Because they said to me: ‘Don’t laugh, talk to her seriously, firmly.’ So, now when I’m telling her something, well, now she understands me, now she pays attention...”

For mothers unaccustomed to verbalizing their frustrations or sadness, it meant becoming emotionally vulnerable and learning to share with their children what they were feeling, as reciprocity is a key tenet of emotional connection:

“I didn’t talk to her about it, it just bothered me and made me angry. I’d tell her ‘let’s go,’ but I didn’t sit like I do here, with her face-to-face, to say ‘why do you do that’… so that’s something I discovered here—that there was another way to solve the problem.” [C]

Mothers described several changes in their child’s behavior as well. A common change was their child’s willingness to engage in communication. D described her child’s progression:

“I had in the house a distant child, poorly behaved, a solitary child; and now I have an affectionate child, who says to me: ‘Mommy, I love you.’ It’s so sweet.”

Children also became more physically expressive, with several mothers sharing that their child newly offered hugs and kisses or asked to be cradled or carried. A explained that her child J initiated physical touch when upset because of the calming session practiced in group:

“And it’s helped her a lot because before she cried and I let her cry, and I didn’t get close to her. But now she looks for me and says she wants a hug, and I give her a hug, I explain how things are, and she understands better.”

Some mothers also noticed an increase in their child’s maturity. Manifestations of maturity included the child being less clingy, more willing to obey, or better able to sustain emotional regulation. “Self-psychology,” as pioneered by the prominent psychoanalyst Heinz Kohut, focuses on the mother unidirectionally nurturing her child and overlooks the value of reciprocal emotional expression and connection. In contrast, MCEP focuses on mother–child co-regulation, the process by which the mother and child mutually engage each other in pursuit of emotional connection. Improved child behavior ensues from this “optimal” mutual emotional connection, as D describes:

“Before he was very impatient. Now he is calm, he says please, he picks up his toys, he’s more organized.”

This supports the view of temperament as a flexible “state”—not a “trait”—that is responsive to mother–child autonomic emotional connection. The increased verbal and physical expression within mother–child dyads led several mothers to feel they were more emotionally connected to their children after participating in MCEP. Evidence of this connection as described by the mothers included children exhibiting awareness of their mothers’ feelings and their own role in positively or negatively affecting those feelings:

“…later, she asked me: ‘Does that make you happy, mommy?’ and I said to her: ‘Yes, that would make me very happy,’ ‘Okay, I will do it for you.’’ [E]

In many cases, observed changes in the child’s behavior directly resulted from the practice of emotional exchange. After D’s apology to B, she noticed a change in the way he responded to her. Before, he was impatient, “And now, no. He stays until I tell him.” B is also more loving, and he continues to seek out close contact:

“B has changed a lot, a lot, a lot. B would go sit in an armchair and he was there and I was here [pointing to two representative spots far away from each other]. And when I was watching television, he would say: ‘No mommy, you look at your phone, don’t watch television, look at your phone’... And now he comes and he lays down on my chest and we can watch television together.”

Interactions like these empowered mothers to better share and respond to their children’s emotions and to their own.

Many mothers expressed that participating in MCEP increased their confidence in their capacities as mothers, which improved their opinions of themselves as people. For some, greater self-confidence came from learning to speak more effectively to their children. For others, witnessing fellow mother–child dyads interacting helped them feel less alone in their parenting challenges and reassured them of their abilities. For C, the group sessions impacted her identity beyond motherhood. After describing herself as “timid” and “insecure,” she expressed that “in group it feels good to be here, it was like I already knew all of you…I felt confident, I didn’t feel afraid…” [Interview 1].

Several mothers described how group sessions impacted their well-being. D stated she felt “happy because I think I’m on the way to preparing myself to be a better mother.” Some mothers discussed their enhanced well-being in relation to their child’s improved behavior. A, for example, described the physical and psychological toll of her child J’s behavior prior to MCEP:

“Before with the stress and all that, my head hurt, I was tired, like depression… But now during these eight sessions, my life has changed…to something better.”

For a few mothers, these psychological and emotional changes impacted their lives beyond motherhood. A reported feeling more relaxed at work. D described increased self-care practices. C felt “more confident.” Mothers even reported friends, family, coworkers, or other people in their lives told them they looked “happier,” [G, D] and “not stressed [G].”

## Discussion

This qualitative analysis answered the two guiding questions of 1) the impact of MCEP on the mother–child pair in the mother’s own words and 2) MCEP alignment with calming cycle theory. By giving voice to participants’ experiences of MCEP, and directly observing MCEP sessions, we answered the question of how parental experience of MCEP aligns with calming cycle theory. On the dyad level, face-to-face calming position and emotional expression synergistically fostered emotional connection. “Barriers” to connection were surmountable; in fact, they were springboards into the calming cycle. On the group level, fellow participants and nurture specialists helped mothers progress through the calming cycle.

The benefit of a group parenting intervention is well-documented [9, 11, 12], but this is the first known study to apply a group framework to an intervention centered on experiential, autonomic mother–child emotional connection in a school setting. For those dyads not initially connected, socioeconomic variables did not affect the intervention in its present context. MCEP might be one of the “low-cost, light-touch behavioral interventions” advocated for by developmental psychologists Kalil & Ryan (2020) [34] that help “narrow income-based parenting gaps.” These observations demonstrate how MCEP can progress the child, mother, and mother–child relationship following group participation.

This study also raises questions for further investigation. Would D have been able to improve her relationship with B if she remained depressed? Does emotional connection relieve depression? In our preterm RCT, mothers in the intervention group reported significantly fewer symptoms of depression and anxiety at four months [35]. Studies have investigated maternal depression’s effect on early childhood “attachment” [36, 37], but no studies have investigated this question in terms of emotional connection. The relationship between clinical depression and MCEP could be explored further in this at-risk mother-preschooler population.

Future studies could explore “ideal” group size for facilitating emotional expression. Studies have shown that just the act of being in a study benefits participants [38]. The fact that the attendance of dyads in this sub-study—as a group, there were only two absences of 64 sessions—was higher than other groups in the RCT, raises the possibility that interactions with the observer motivated attendance (Table 2). Mothers as uniquely effective peer supports—establishing trust through shared experiences—raises the prospect of previous participants becoming nurture specialists, like a community health worker model [39, 40]. Mothers reported that this format offers much-needed validation and reassurance they may not otherwise receive. Social isolation also emerged as a sub-theme in the findings. These two factors suggest strategies should be considered to connect MCEP recipients with the preschool’s social integration services, and other community resources, to build upon social support offered through group sessions.

**Table 2:** Dyad attendance

**Mother/Child**	**1** 2/19	**2** 2/26	**3** 3/5	**4** 3/12	**5** 3/19	**6** 3/26	**7** 4/2	**8** 4/9	**9** 4/16	**10** 4/23	**11** 4/30	**12** 5/7	**13** 5/14	**14** 5/21	**15** 5/28	**Total**
G/M	X	X	X	X	X	X		X	X							8
C/N	X		X	X	X	X	X	X	X							8
K/S	X	X	X	X		X	X	X		X						8
E/H	X	X	X	X	X		X		X		X					8
A/J		X		X	X	X				X	X		X			7
D/B		X	X	X	X	X	X				X		X			8
F/R		X	X				X			X			X	X	X	7
L/K				X	X	X		X				X	X	X	X	8

This study generated two codebooks that can be applied to other data, enabling retesting and expansion of our findings.

### Limitations

A key limitation to this study is sample size. With only eight dyads, sociodemographic representation and diversity of perspectives are limited. Participating children’s perspectives were not included. This limitation was recognized and accounted for by avoiding overgeneralization of participant experiences and including each participating mother’s voice in the presentation of study findings.

Other limitations are inherent to qualitative research, especially the subjectivities of the qualitative research team. The researchers’ backgrounds and points of view may have influenced analyses. The fact that ESM conducted all observations and interviews may have skewed subjectivity further. Fluency may have also been a limitation; ESM and MCM, although fluent, are not native Spanish-speakers. We therefore used the tenets of credibility, authenticity, criticality, and integrity to overcome bias, as discussed in the Methods section.

Also, this study cannot answer the question of required intervention “dosage,” since only two dyads missed one of their eight sessions (Table 2). Thus, a clear dose–response relationship like Muzik et al. (2015)’s Mom Power intervention cannot be determined [41].

It is important to note that fathers, although welcome to attend sessions, did not do so. A future direction is studying father-child and/or father-mother–child pairs.

## Conclusions

MCEP fills a gap in early childhood education services, particularly within programs operating on narrow margins and serving families with limited access to health services. Because MCEP can be administered entirely by school personnel, it is potentially more sustainable and cost-effective than current programs focused on the child, the mother, or the pair in a healthcare setting. This study has demonstrated the alignment of MCEP with calming cycle theory and explored the intervention’s impact through observations of participants’ interactions during group and through mothers’ reflections during interviews. It was striking that the data so strongly supported the synergy of two of the foundational elements of the calming cycle: emotional expression and the face-to-face on lap calming position (Fig. 4). Further, emotional expression to reach emotional connection overcomes all barriers, especially with the help of group and nurture specialist facilitation. The study team collectively witnessed the transformative power of MCEP for the mother–child participants. Our analyses are complementary to quantitative results on the full 90-dyad RCT.

Taken together with the quantitative data, hearing the words of mothers in this qualitative sub-study calls for implementation studies towards broad dissemination of MCEP into preschools.

### Supplementary Information

**Additional file 1: Supplement 1.** Unstructured Interview Guide.

**Additional file 2: Supplement 2.** Observation code co-occurrence (expansion of Fig. 3).

**Additional file 3: Supplement 3.** Selected frequency of observation codes per session (expansion of Fig. 4).

## Data Availability

The datasets generated and/or analyzed during the current study are not publicly available due to research ethics board restrictions but are available from the corresponding author on reasonable request.

## Abbreviations

CLC: Children’s Learning Centers of Fairfield County
MCEP: Mother-Child Emotional Preparation Program
RCT: Randomized controlled trial
SC: Standard Care
WECS: Welch Emotional Connection Screen

## Footnotes

1 *Denotes first use of initial of participant pseudonym.
