# Supplementary material for: Qualitative insights from a randomized clinical trial of a mother–child emotional preparation program for preschool-aged children
Source: BMC Psychol. 2023 Sep 1;11:257. doi: 10.1186/s40359-023-01288-y (PMC10472558; doi:10.1186/s40359-023-01288-y)
Supplement: Supplementary file 1 — Additional file 1: Supplement 1. Unstructured Interview Guide. [file 40359_2023_1288_MOESM1_ESM.docx]

**Supplement 1**

Note: Interviews were conducted with the intervention labeled as Family Nurture Intervention, but the intervention was subsequently renamed MCEP.
