# Supplementary material for: Qualitative insights from a randomized clinical trial of a mother–child emotional preparation program for preschool-aged children
Source: BMC Psychol. 2023 Sep 1;11:257. doi: 10.1186/s40359-023-01288-y (PMC10472558; doi:10.1186/s40359-023-01288-y)
Supplement: Supplementary file 2 — Additional file 2: Supplement 2. Observation code co-occurrence (expansion of Fig. 3). [file 40359_2023_1288_MOESM2_ESM.docx]

**Supplement 2**. Observation code co-occurrence (expansion of Figure 3)

|  | **1.**  Calming | **2.**  Emotional expression | **3.**  Crying | **4.**  Vocal commun-ication | **5.**  Facial commun-ication | **6.**  Group impact | **7.**  Calming position | **8.**  Nurture specialist | **9.**  Physical affection |
| --- | --- | --- | --- | --- | --- | --- | --- | --- | --- |
| **A.** Opposition | 1 | 17 | 7 | 2 | 2 | 5 | 20* | 13 | 1 |
| **B.** Emotional expression | 8 | - | 20* | 26* | 14 | 12 | **43***** | 36** | 12 |
| **C.**  Calming position | 12 | **43***** | 12 | 8 | 22* | 14 | - | 40** | 25* |

Notes: co-occurrence * = 20 – 29 times, ** = 30 – 39 times, ***40 or more times
