# Supplementary material for: Qualitative insights from a randomized clinical trial of a mother–child emotional preparation program for preschool-aged children
Source: BMC Psychol. 2023 Sep 1;11:257. doi: 10.1186/s40359-023-01288-y (PMC10472558; doi:10.1186/s40359-023-01288-y)
Supplement: Supplementary file 3 — Additional file 3: Supplement 3. Selected frequency of observation codes per session (expansion of Fig. 4). [file 40359_2023_1288_MOESM3_ESM.docx]

**Supplement 3**. Selected frequency of observation codes per session (expansion of Figure 4)

|  | Barriers to connection | Soothing | Discipline | Emotional expression | Facial communication | Group impact | Calming position | Nurture specialist | Physical affection |
| --- | --- | --- | --- | --- | --- | --- | --- | --- | --- |
| 1 | 6 | 2 | 2 | 2 | 2 | 4 | 5 | 16** | 4 |
| 2 | 5 | 5 | 1 | 5 | 5 | 4 | 13* | 24*** | 2 |
| 3 | 10 | 4 | 1 | 6 | 4 | 3 | 11* | 13* | 4 |
| 4 | 10 | 1 | 2 | 15* | 5 | 5 | 17** | 18** | 3 |
| 5 | 4 | 2 | 1 | 10 | 2 | 10 | 15 | 12* | 1 |
| 6 | 6 | 1 | 2 | 15* | 1 | 11* | 10 | 18** | 2 |
| 7 | 9 | 0 | 1 | 10 | 2 | 10 | 11* | 15* | 2 |
| 8 | 6 | 0 | 1 | 7 | 2 | 13* | 8 | 8 | 3 |
| 9 | 6 | 1 | 1 | 7 | 2 | 6 | 6 | 11* | 1 |
| 10 | 4 | 0 | 2 | 4 | 2 | 5 | 11* | 6 | 7 |
| 11 | 8 | 4 | 2 | 10 | 0 | 1 | 8 | 11* | 3 |
| 12 | 9 | 1 | 3 | 6 | 4 | 10 | 15* | 12* | 3 |
| 13 | 2 | 0 | 1 | 1 | 3 | 3 | 7 | 8 | 3 |
| 14 | 3 | 1 | 2 | 7 | 2 | 5 | 5 | 8 | 3 |
| *Total* | 88 | 22 | 22 | 105 | 36 | 90 | 142 | 180 | 41 |

Notes: frequency * = 11-15, ** = 16-20, *** = >20
